# Innate IFN-γ ameliorates experimental autoimmune encephalomyelitis and promotes myeloid expansion and PDL-1 expression

**DOI:** 10.1038/s41598-017-18543-z

**Published:** 2018-01-10

**Authors:** Madeleine P. J. White, Gill Webster, Faith Leonard, Anne Camille La Flamme

**Affiliations:** 1 School of Biological Sciences, Victoria University of Wellington, Wellington, New Zealand; 2 Innate Immunotherapeutics, Auckland, New Zealand; 3 Malaghan Institute of Medical Research, Wellington, New Zealand

## Abstract

The innate immune system plays a central role in the immune-mediated pathology of multiple sclerosis, and is a therapeutic target for progressive disease. Recently, it has been demonstrated that MIS416, a novel immunomodulatory microparticle that activates NOD-2 and TLR-9-signaling, has disease-modifying activity in multiple sclerosis models. This activity is dependent on innate IFN-γ; however, the precise immune regulatory mechanisms amplified by MIS416 have not previously been determined. Using the experimental autoimmune encephalomyelitis model, MIS416 treatment was associated with IFN-γ–dependant expansion of Treg number and increased suppressive function; however, these cells did not account for disease reduction. Additionally, MIS416 treatment stimulated increased nitric oxide production that was IFN-γ–dependant but dispensable for protection. Finally, MIS416-mediated protection was shown to correlate with IFN-γ–dependant expansion of PDL-1-expressing peripheral myeloid cells, a subset of which was found to be selectively recruited to the brain. This central nervous system trafficking was independent of neuro-inflammatory signals as it occurred in MIS416-treated healthy mice. Together, these findings provide insight into regulatory myeloid cell activities amplified by MIS416-mediated NOD-2 and TLR-9 signalling and highlight the potential importance of these cells in accessing the brain where they may act locally and contribute to the control of neuroinflammation.

## Introduction

Multiple sclerosis (MS) is a neuroinflammatory disease with an autoimmune component that is characterised by activation of self-reactive lymphocytes, which enter the central nervous system (CNS) and cause destruction of myelin producing cells and neurons leading to the formation of inflammatory lesions. A number of immune-modifying therapies are now available to treat the relapsing-remitting form of MS, most of which target the peripheral immune system. Unfortunately, such treatments are largely ineffective in patients with the secondary progressive form of MS (SPMS), supporting the hypothesis that CNS-compartmentalised, innate inflammation is the key driver of SPMS pathogenesis, which appears to be independent of peripheral adaptive immunity. Therefore, to treat progressive MS, new therapeutic strategies are required that have direct anti-inflammatory activity within the CNS and restore CNS homeostasis.

Modification of the innate immune system with MIS416, a TLR9 and NOD2 agonist derived from *Propionibacterium acnes* ^1^, is currently under investigation as a treatment for SPMS. Previous studies have shown that MIS416 has a good safety profile in SPMS patients and works effectively as an immunomodulator^2,3^. Using the experimental autoimmune encephalomyelitis (EAE) model of MS, it has been shown that treatment with MIS416 significantly reduced disease burden in EAE mice when treated prophylactically or therapeutically^4^. MIS416 treatment induced rapid production of high levels of IFN-γ in the serum of treated mice and SPMS patients^4^, and IFN-γ was found to be essential for disease protection in EAE^4^. Whilst IFN-γ is typically considered to be a pro-inflammatory product of effector T cells, it has been also been demonstrated that IFN-γ^−/−^ mice develop more severe EAE than wild type (WT) controls^5^, highlighting alternate, IFN-γ–dependant negative feedback mechanisms that can constrain EAE disease activity. This study aimed to determine the mechanisms by which MIS416-induced IFN-γ reduces disease severity in EAE and investigate the IFN-γ-dependent effects on disease-inducing auto-reactive CD4 T cells as well as the peripheral and CNS resident myeloid cells that are directly targeted by MIS416.

## Methods

### Animals

Female C57BL/6, B6.SJL-ptprca, 2D2 (MOG_35-55_ TCR transgenic)^6^, and IFN-γ-deficient mice between the ages of 8–12 weeks old were obtained from the Malaghan Institute of Medical Research. All experimental procedures in this study were approved by the Victoria University of Wellington Animal Ethics Committee (protocol 2011-R21 and 2014-R23), and all experiments were performed in accordance with the relevant guidelines and regulations.

### EAE induction and treatments

Mice were immunized by subcutaneous (s.c.) injection in each hind flank with an emulsion consisting of 50 μg MOG_35-55_ peptide (Genscript, Piscataway, NJ) and 500 μg heat-inactivated *Mycobacterium tuberculosis* (Difco Laboratories, Detroit, USA) in incomplete Freund’s adjuvant (Sigma, St. Louis, MO). In addition, mice received 200 ng pertussis toxin (Sapphire Bioscience, Redfern, NSW, Australia) intraperitoneally (i.p.) on days 0 and 2 post-immunization (p.i.) as previously described^7^. Mice were weighed and monitored for signs of disease daily. EAE disease was scored 0-5 as follows: 0 = unaffected, 0.5 = loss of tonicity in distal region of tail, 1 = half-tail paralysis; 2 = full tail paralysis; 3 = one hind limb paralysis or severe weakness in both hind limbs; 4 = full hind limb paralysis; and 5 = moribund^8,9^. MIS416 was provided by Innate Immunotherapeutics (Auckland, New Zealand) and was administered (100 μg/mouse) weekly via the tail vein. Aminoguanidine hemisulfate (Sigma) was administered to mice (100 mM in drinking water) to inhibit inducible nitric oxide synthase (iNOS) *in vivo* ^9^.

### Isolation and in vitro culture of cells

A single cell suspension was prepared from spleen and lymph nodes as previously described^10^, and the number of viable cells were counted based on trypan blue dye exclusion. CD4 T cells were isolated using Dynabeads Untouched mouse CD4 Kit (Life Technologies, USA) and Treg were isolated using MACs CD4+ CD25+ mouse regulatory T cell isolation kit (Miltenyi Biotec, Cologne, Germany) as per manufacturer’s instructions.

Splenocytes (1 × 10^6^ cells/well) and CD4 T cells (1 × 10^5^ cells/well) were cultured in culture media containing Dulbecco’s minimal essential medium, 10% FCS, 10 mM Hepes, 100 U/ml penicillin plus 100 μg/ml streptomycin, 2 mM L-glutamine, and 50 μM 2-mercaptoethanol (all from Invitrogen, Carlsbad, CA). In addition, cells were cultured with MOG peptide (27 μg/ml), MIS416 (20 μg/ml), concanavalin A (ConA, 1 μg/ml) or lipopolysaccharide (LPS, 200 ng/ml) in the absence or presence of the iNOS inhibitor, aminoguanidine (200 mM)) for 48-72 hours. Previously published dose-response curves identified that these doses give reliable and reproducible results^1,11^. For proliferation assays, isolated CD4 T cells and splenocytes were stained with 5 μM CFSE as previously described^12^. CD4 T cells were stimulated with 2.5 × 10^4^ anti-CD3/CD28 T cell activator beads (Life Technologies, USA) for 72 hours.

### Cytokine analysis

Murine IFN-γ and IL-10 in culture supernatants were analyzed using a sandwich ELISA. All reagents were purchased from Becton Dickinson Biosciences and used as per the manufacturer’s instructions. Nitric oxide (NO) was measured in culture supernatants using the Griess reaction as previously described^13^ and NO levels in the serum were detected using a Nitric Oxide Detection kit (Enzo Life Sciences, Farmingdale, New York) according to the manufacturer’s instructions.

### Flow cytometry

Cells isolated from spleen, lymph nodes, brain, and spinal cord were analysed using a FACS Canto II flow cytometer (BD, Franklin Lakes, NJ) as described^14^. The following antibodies were used for phenotypic analysis: mouse anti-NK1.1 IgG2a, hamster anti-CD11c IgG, rat anti-Ly6C IgG2c, rat anti-Ly6G IgG2a, mouse anti-FoxP3 IgG1, mouse anti-CX3CR1 IgG2a, and mouse anti-CD45.1 IgG2a from Biolegend (San Diego, CA) and rat anti-CD4 IgG2a, rat anti-CD8α IgG2a, rat anti-CD25 IgG1, rat anti-I-A^b^ (MHC-II) IgG2a, rat anti-CD11b IgG2b, mouse anti-CD45.2 IgG2 and rat anti-Gr1 IgG2b from BD Biosciences (San Jose, CA) and rat anti-PD-L1 IgG2a and rat anti-F4/80 IgG2a from eBiosciences (San Diego, CA). Fcγ receptors were blocked by the addition of purified anti-CD16/32 antibodies (Fc Block; 1 μg/10^6^ cells; BD Biosciences) 15 minutes prior to surface staining. For intracellular cytokine staining, GolgiStop (1 μg/10^6^ cells; BD Biosciences), ionomycin (500 ng/ml; Sigma) and PMA (50 ng/ml) were added to the Con A-stimulated splenocyte cultures 5 hours prior to surface antibody staining. After extracellular staining, cells were fixed and permeabilized using Transcription Factor Buffer (BD Biosciences, USA) and stained with rat anti-IFN-γ IgG1 (BD Biosciences) following the manufacturer’s instructions and analyzed using BD FACS Canto II flow cytometer (BD Biosciences).

### *In vivo* proliferation

Single cell suspensions were prepared from the spleen and lymph nodes of 2D2 mice and CFSE-labelled as indicated previously^7^. CFSE-labelled 2D2 cells (10 × 10^6^) were injected i.v. into B6.SJL-ptprca mice, and the following day mice were immunized for EAE and treated with MIS416 as previously described^4^. Mice were culled 5 days later and the spleen, blood and draining lymph nodes were processed and analyzed by flow cytometry to assess 2D2 CD4 T cell proliferation, using rat anti-CD45.2 and rat anti-CD4 antibodies to clearly identify donor CD4 T cells.

### Statistical analyses

All data were graphed and analysed using GraphPad Prism version 7 (La Jolla, CA, USA). In general, for two group comparisons, unpaired or paired Student’s t test was used for parametric data, and Mann-Whitney for non-parametric data. For >2 groups, one-way or two-way ANOVA was used with the recommended multiple comparison tests as indicated in the figure legend and as recommended by GraphPad Prism. Differences of p < 0.05 were considered significant.

## Results

### MIS416 reduced disease severity of EAE model and led to an increase in splenic T cell populations

**Figure 1:**
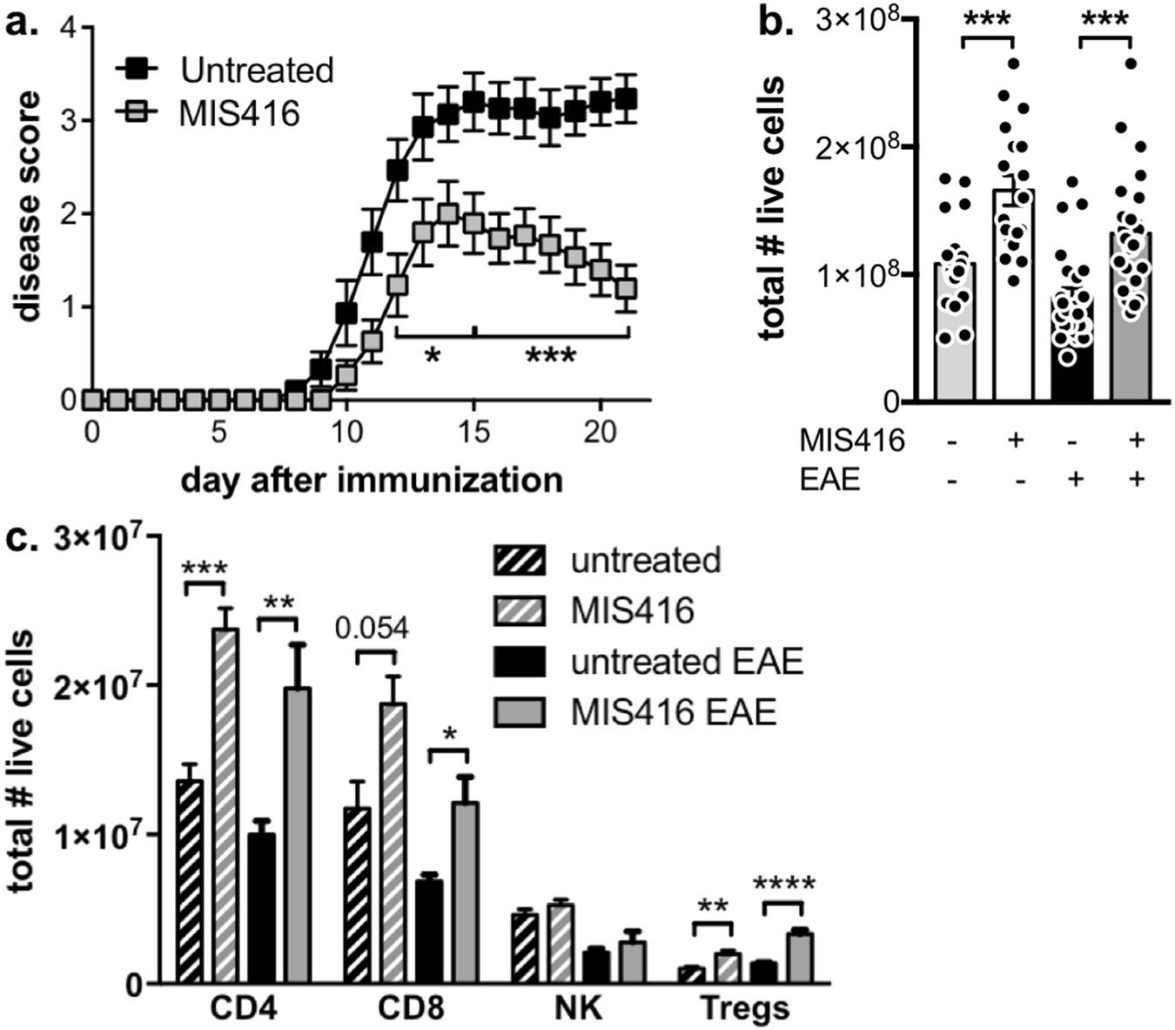
MIS416 administration reduced EAE severity and led to an expansion in splenic T cell populations. (**a**) C57BL/6 mice were treated with MIS416 weekly (i.v.; 100 μg/mouse) starting on day 0, immunized to induce EAE, and scored daily for EAE (0–5). Shown are the means and SEM of individual mice (n = 15/group) from 3 experiments. ***p < 0.001 and *p < 0.05 by two-way ANOVA with Sidak’s multiple comparisons test. (**b**,**c**) MIS416 increased the total number of splenocytes, CD4 T cells, CD8 T cells, and Treg in EAE mice 22 days post-immunization and healthy mice 15 days post treatment initiation. Total splenocytes were assessed by trypan blue dye exclusion and immune subsets by flow cytometry. Gating strategy is found in Suppl Fig. 1. Shown are the means and SEM of individual mice (**b**, n = 18–25/group from 5 experiments; **c**, n = 5–15/group from up to 3 experiments). ****p < 0.0001, ***p < 0.001, **p < 0.01, and *p < 0.05 by one-way ANOVA with Tukey’s multiple comparisons test.

As shown in previous studies^4^, weekly treatment with 100 μg MIS416 i.v. starting on the day of immunization effectively reduced disease severity in the EAE model (Fig. 1a). Analysis of T cell subsets in secondary lymphoid tissue (spleen) on day 22 after disease induction showed a significant increase in the total number of splenocytes in MIS416-treated mice (Fig. 1b). A similar increased was found in healthy MI416-treated mice 15 days after treatment initiation (Fig. 1b). This increase in splenocyte numbers in both EAE and healthy MIS-treated mice was in part due to increased numbers of CD4 and CD8 T cells, as well as Tregs, whilst numbers of NK cells were not significantly altered (Fig. 1c and Suppl Fig. 1).

### CD4 T cell proliferation was reduced by MIS416 and was dependant on accessory cells

**Figure 2:**
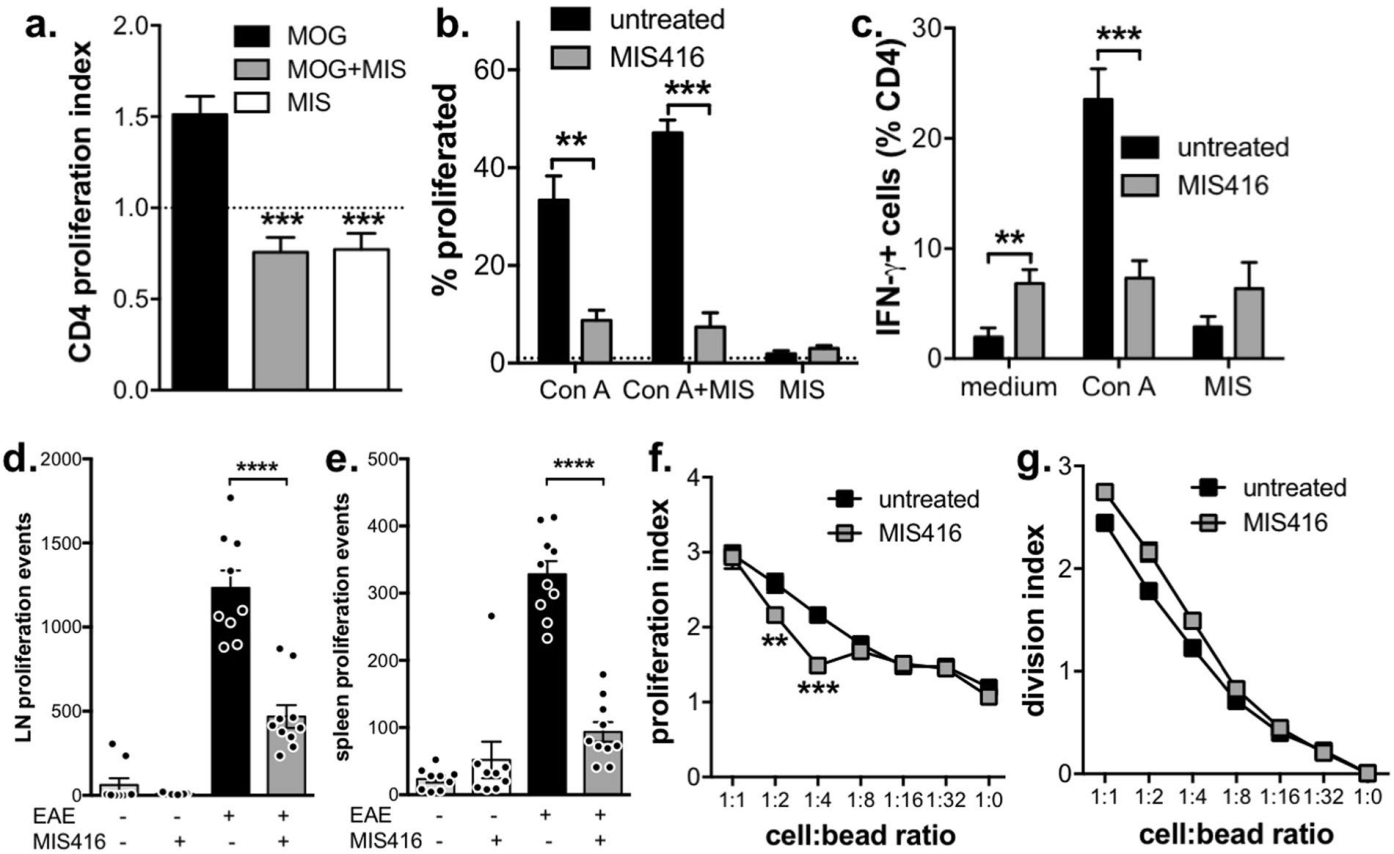
CD4 T cell proliferation was reduced by MIS416 administration in vitro or in vivo but only when APC were present. (**a**) Splenocytes (1 × 10^6^/ml) were isolated from EAE mice that had been immunized 21 days previously, labelled with CSFE, and stimulated *in vitro* with MOG (27 μg/ml), MIS416 (20 μg/ml), or both for 72 hours. The dotted line represents proliferation in wells with medium alone. Shown are the means and SEM of proliferative indexes from individual mice (n = 4) from one experiment. ***p < 0.001 compared to MOG by one-way ANOVA with Tukey’s multiple comparison test. (**b**,**c)**. Splenocytes (1 × 10^6^/ml) were isolated from healthy mice treated with MIS416 15 days previously, labelled with CSFE, and stimulated *in vitro* with Con A (3 μg/ml), MIS416 (20 μg/ml), or both for 48 hours. The % of CD4^+^ cells that proliferated (**b**) and were IFN-γ^+^ (**c**) was quantified by flow cytometry. Shown are the means and SEM of individual mice (n = 8/group; 4 for Con A + MIS) from 2 independent experiments. ***p < 0.001 and **p < 0.01 by two-way ANOVA with Sidak’s multiple comparison test. (**d**,**e**) CFSE-labelled 2D2 cells were transferred to congenic mice (CD45.1) one day before MIS416 treatment and EAE immunization. After 5 days, proliferation of CD45.2 2D2 CD4^+^ T cells in the draining lymph node and spleen was determined by flow cytometry (gating strategy shown in Suppl Fig. 2). Shown are the means and SEM of values from individual mice (n = 9–10/group) from two independent experiments. ****p < 0.0001 by one-way ANOVA with Tukey’s multiple comparison test. (**f**,**g**). Purified CD4 T cells were isolated from healthy, untreated and MIS416-treated mice at 24 hour post dose 3 (day 15), labelled with CFSE, and stimulated *in vitro* with anti-CD3/CD28 expander beads at increasing cell:bead ratios for 48 hours. The proliferation (**f**) and division (**g**) indexes were measured by flow cytometry. Shown are the means and SEM of duplicate wells from one experiment. ***p < 0.001 and **p < 0.01 by two-way ANOVA with Sidak’s multiple comparison test.

Although MIS416 has been shown to reduce antigen-specific cytokine production during EAE^4^, it is not known how MIS416 affects CD4 T cell proliferation *in vitro* or *in vivo*. To determine the *in vitro* effect of MIS416 on CD4 T cell proliferation, MIS416 was added to MOG-stimulated splenocyte cultures from EAE immunized mice, and was shown to suppress MOG antigen-induced CD4 T cell proliferation (Fig. 2a). MIS416 added to cultures in the absence of antigen showed that MIS416 on its own did not support CD4 T cell proliferation (Fig. 2a). Furthermore, splenocyte cultures from healthy mice treated with MIS416 demonstrated reduced T cell proliferation as well as a decreased number of IFN-γ^+^ CD4 T cells in response to antigen-independent stimulation by mitogen (ConA), when compared to untreated, healthy mice (Fig. 2b,c). Paradoxically, the number of IFN-γ^+^ CD4 T cells was significantly enhanced in unstimulated cultures (Fig. 2c) suggesting an *in vivo* enhancement.

To determine if MIS416 reduced the proliferative capacity of CD4 T cells *in vivo*, CFSE-labelled CD4 T cells from 2D2 mice, the majority of which express a MOG-specific T cell receptor^15^ were injected into congenic (CD45.1) mice prior to EAE immunization. Five days post-immunization, during the period of peak lymphocyte proliferation, we found that MIS416 treatment significantly reduced the number of proliferative events in the lymph nodes and spleens of immunized mice (Fig. 2d,e and Suppl Fig. 2). In contrast, purified CD4 T cells from MIS416-treated and untreated mice showed similar proliferative responses to anti-CD3/CD28 stimulator beads (Fig. 2f,g). A decreased proliferative index was observed at bead ratios of 1:2 and 1:4 but this decrease was not reflected in the division index suggesting that MIS416 treatment was not directly inducing T cell anergy in the absence of other immune cells. Together, these results indicate that MIS416-mediated suppression of CD4 T cell proliferation is dependent on MIS416-associated alterations in the immune environment of the lymphoid tissue.

### MIS416 administration enhanced Treg function in an IFN-γ-independent manner

**Figure 3:**
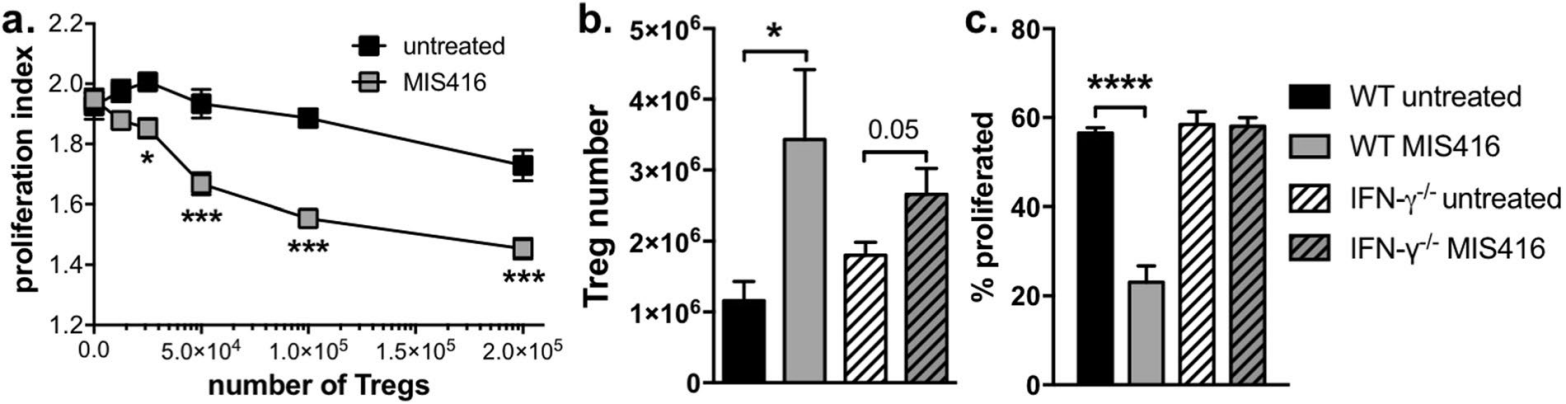
Splenic Treg number and function was enhanced by *in vivo* MIS416 administration but did not appear to correlate with IFN-γ-dependent protection from EAE. (**a**) Treg were isolated from healthy mice and MIS416-treated mice one day post-dose 3 and cultured with CFSE-labelled, anti-CD3/CD28 stimulated CD4+ T cells at increasing Treg ratios. After 72 hours, the CD4 T cell proliferative index was assessed by flow cytometry. Shown are the means and SEM of Treg preparations from individual mice (n = 5–6/group) from 2 independent experiments. ***p < 0.001 and *p < 0.05 by two-way ANOVA with Sidak’s multiple comparison test. (**b**) MIS416 increased the total number of Treg in healthy WT and IFN-γ^−/−^ mice 15 days post treatment initiation. Treg were identified as CD25^+^FoxP3^+^CD4^+^ cells by flow cytometry. Shown are the means and SEM of individual mice (n = 7/WT group and n = 12/IFN-γ^−/−^ group) from 3 experiments. *p < 0.05 by one-way ANOVA with Tukey’s multiple comparisons test. (**c**) Splenocytes were isolated from WT and IFN-γ^−/−^ mice one day post the third dose of MIS416 (day15), labelled with CSFE, and cultured *in vitro* (1 × 10^6^/ml) with Con A (3 μg/ml) for 48 hours. The % of CD4^+^ cells that proliferated was quantified by flow cytometry. Shown are the means and SEM of individual mice (n = 12/group) from 3 independent experiments. ****p < 0.0001 by one-way ANOVA with Tukey’s multiple comparison test.

Given the significant increase in Treg number by MIS416 treatment (Fig. 1c) and the known capacity of Treg to suppress T cell proliferation, we investigated whether MIS416 treatment also enhanced Treg function. We found that purified splenic Treg from MIS416-treated healthy mice were more effective at suppressing CD4 T cell proliferation *in vitro* than those purified from untreated mice (Fig. 3a) indicating that MIS416 enhanced Treg function. Since IFN-γ is essential for MIS416-mediated suppression of EAE^4^, we assessed whether the enhancement in Treg number occurred in MIS416-treated IFN-γ^−/−^ mice and found that MIS416 was still able to enhance Treg numbers in the absence IFN-γ (Fig. 3b). Although the expansion was not as dramatic as that seen in WT mice, we believe this reduced expansion may, in part, be due to increased numbers of Treg in the spleens of untreated IFN-γ^−/−^ mice. Furthermore, *in vitro* CD4 T cell proliferation was not suppressed in splenocyte cultures from MIS416-treated IFN-γ^−/−^ mice (Fig. 3c) suggesting that despite the increased presence of Treg, these cells are not the major contributors to the effects of MIS416. Together this data suggests MIS416 suppression of CD4 T cell proliferation is dependent on IFN-γ but Treg are not major contributors.

### MIS416 specifically expanded the red pulp macrophage population and upregulated myeloid PDL-1expression in an IFN-γ-dependent manner

**Figure 4:**
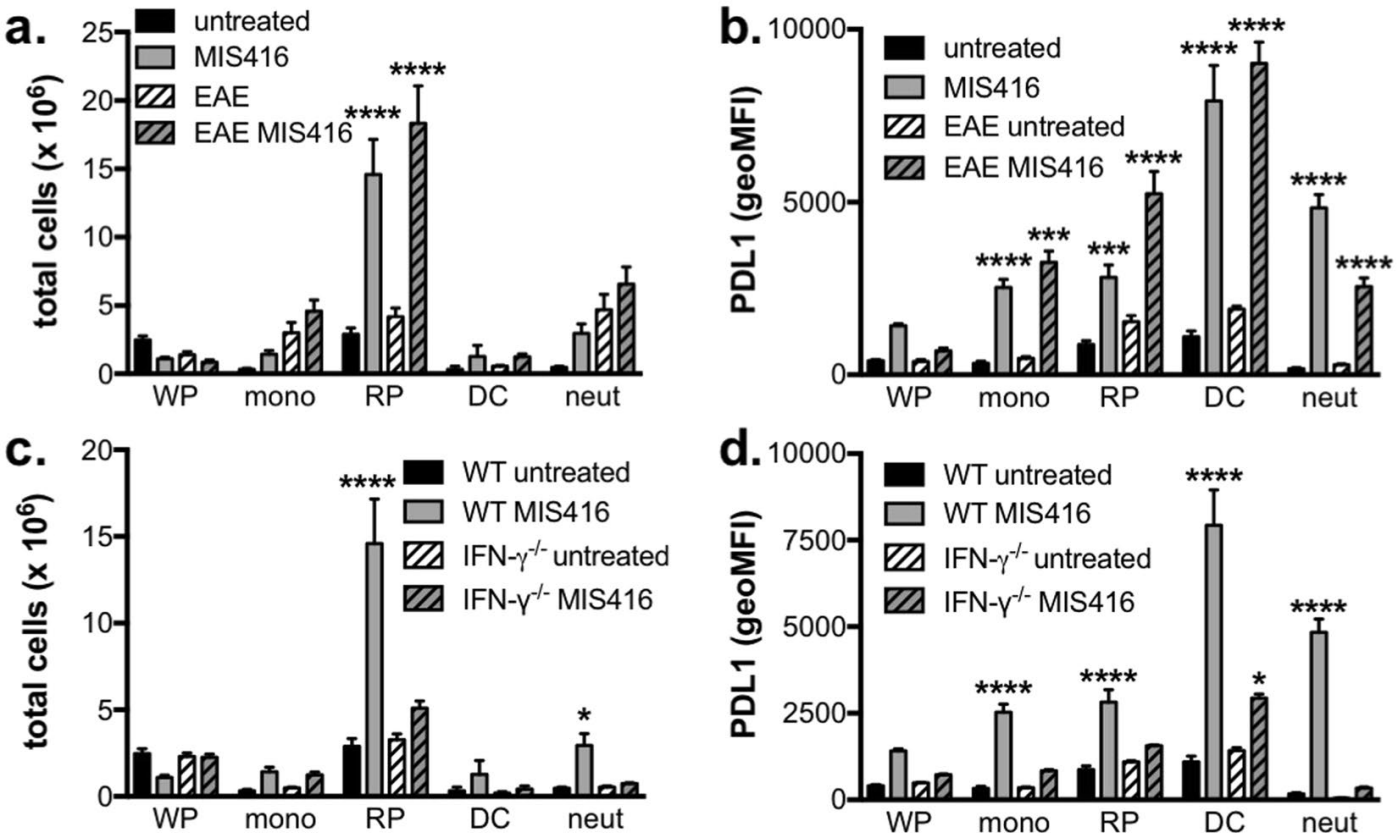
The selective expansion of red pulp macrophages (F4/80+) and increased expression of PDL1 on all splenic myeloid populations by MIS416 required IFN-γ. (**a**,**b**) Splenocytes were isolated from EAE mice on day 22 post immunization and from healthy mice 15 days post MIS416 treatment initiation (24 hours post dose 3), and the total number of each splenic myeloid subset and the expression of PDL-1 (geoMFI) were assessed by flow cytometry (gating strategy in Suppl Fig. 3a). Shown are the means and SEM of individual mice (n = 13–14) from 3 independent experiments. (**c**,**d**) Splenocytes were isolated from healthy WT and IFN-γ^−/−^ mice 15 days post MIS416 treatment initiation and myeloid populations and PDL-1 expression assessed. Shown are the means and SEM of individual mice (n = 14/WT group and 11/ IFN-γ^−/−^ group) from 3 independent experiments. (**a–d**) ****p < 0.0001, ***p < 0.001, **P < 0.01, and *p < 0.05 by one-way ANOVA with Tukey’s multiple comparisons test.

In addition to increasing lymphocyte numbers, MIS416 has also been reported to expand splenic myeloid subsets^4^. To further define these populations and determine whether any changes correlated with disease protection in EAE, we quantified the major splenic myeloid populations as follows: neutrophils (Gr-1^high^, Ly6G^+^, CD11b^+^, SSC^high^), white pulp macrophages (Gr-1^−^, CD11b^+^, F4/80^−^), red pulp macrophages (Gr-1^−^, CD11b^−^, F4/80^+^), splenic CD11c dendritic cells (Gr-1^−^, CD11c^+^, CD11b^+/−^, F4/80^+/−^), and infiltrating monocytes (Gr-1^low^, Ly6C^+^, CD11b^+^). Using the gating strategy in Supplementary Figure 3a, it is evident that MIS416 administration significantly increased the number of red pulp macrophages in healthy and EAE immunized mice (Fig. 4a). While neutrophil numbers were increased by MIS416 treatment in healthy mice, these did not increase further during EAE (Fig. 4a). Although only red pulp macrophage numbers increased, MIS416 treatment upregulated PDL-1 and MHC class II expression on all splenic myeloid subsets analysed in unimmunized and immunized mice (Fig. 4b and Suppl Fig. 3b). Moreover, the absence of IFN-γ abolished red pulp macrophage expansion as well as the upregulation of PDL-1 and MHC class II (Fig. 4c,d and Suppl Fig. 3b) indicating that these changes are correlates of IFN-γ induced by MIS416. In contrast, although PDL-1 expression was modestly upregulated on lymphocytes, this upregulation was not abolished by IFN-γ-deficiency (Suppl Fig. 4) further supporting the myeloid-specificity of MIS416-mediated effects.

### MIS416-stimulated NO was responsible for the suppression of CD4 T cell proliferation but not disease protection

**Figure 5:**
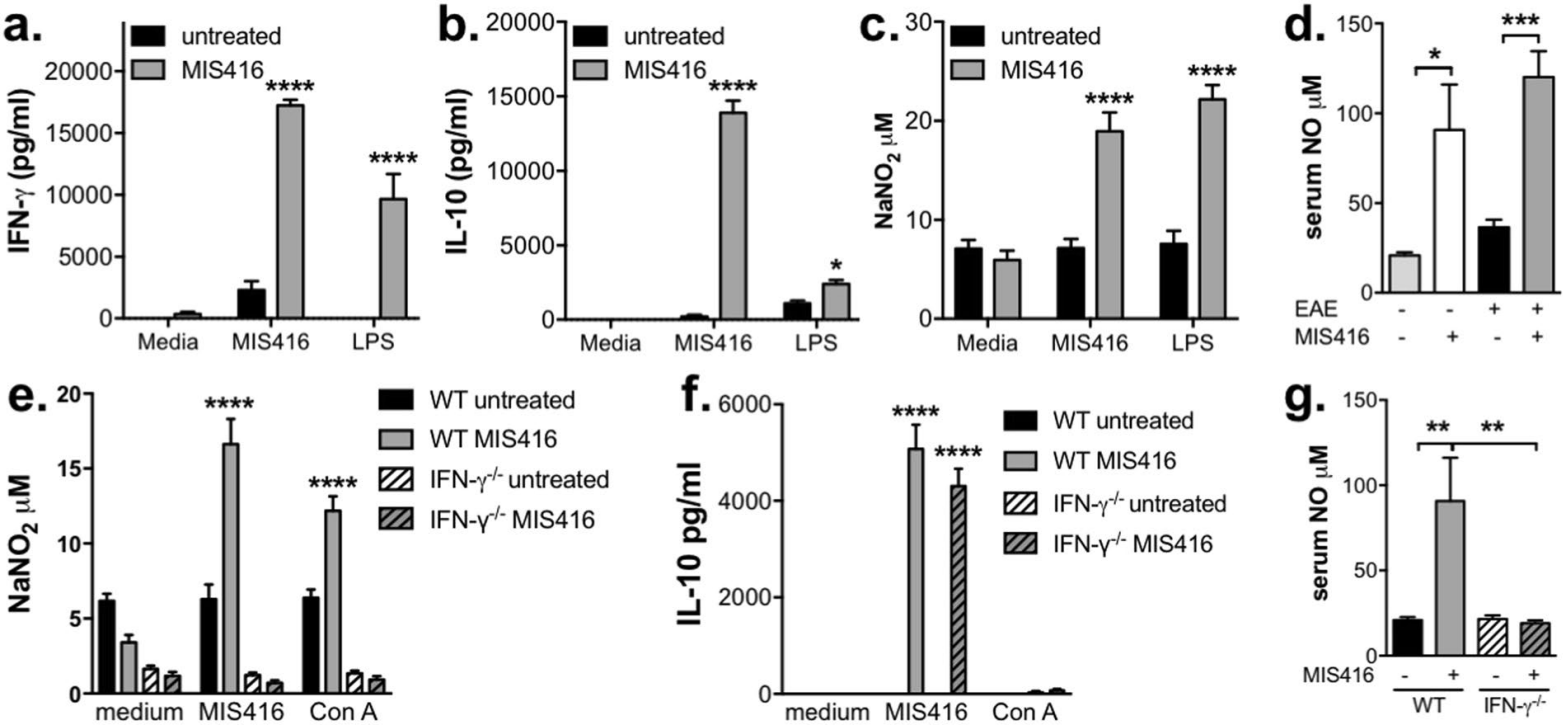
MIS416 promoted the production of NO but not IL-10 in an IFN-γ-dependent manner. (**a–c**) Splenocytes were isolated from healthy mice 15 days post MIS416 treatment initiation and stimulated (1 × 10^6^/ml) with MIS416 (20 μg/ml) or LPS (200 ng/ml). After 24 hours, the amount of IFN-γ (**a**), IL-10 (**b**), and NO (**c**) was measured in the culture supernatants. Shown are the means and SEM from individual mice (n = 8-10 mice/group) from 2 experiments (medium & MIS416 stimulation) or 5 mice/group from 1 experiment (LPS stimulation). ****p < 0.0001 and *p < 0.05 by two-way ANOVA with Sidak’s multiple comparison test. (**d**) MIS416 increased serum levels of NO in healthy mice 15 days post MIS416 treatment initiation and in EAE mice on day 21 post immunization. Shown are the means and SEM from individual mice (n = 9–13/group) from 2 (healthy) or 3 (EAE) experiments. ***p < 0.001 and *p < 0.05 by one-way ANOVA with Tukey’s multiple comparisons test. (**e**,**f**) Splenocytes were isolated from healthy WT and IFN-γ^−/−^ mice 15 days post MIS416 treatment initiation. Splenocytes were stimulated with MIS416 (20 μg/ml) or Con A (3 μg/ml) for 48 hours and NO and IL-10 quantified in the culture supernatant. Shown are the means and SEM of individual mice (n = 5/WT group and 12/ IFN-γ^−/−^ group) from 2–3 independent experiments. ****p < 0.0001 by two-way ANOVA with Sidak’s multiple comparison test. (**g**) Serum NO levels were assessed in healthy WT and IFN-γ^−/−^ mice 15 days post MIS416 treatment initiation. Shown are the means and SEM of individual mice (n = 8–10/group) from 2 independent experiments. **p < 0.01 by one-way ANOVA with Tukey’s multiple comparisons test.

In light of MIS416-mediated expansion of splenic macrophages, we investigated their functional status by determining responsiveness to *ex vivo* innate stimulation with both MIS416 as well as the TLR4 ligand, LPS. Stimulation of splenocytes from MIS416-treated mice with MIS416 or LPS resulted in significantly higher levels of NO, IFN-γ and IL-10 in culture supernatants compared to those from healthy animals (Fig. 5a–c). Consistent with this, serum levels of NO were significantly elevated after MIS416 treatment in EAE as well as healthy mice (Fig. 5d). Additionally, the MIS416-associated increased NO production *in vitro* and *in vivo* was abolished in the absence of IFN-γ (Fig. 5e,f) whilst *in vitro* IL-10 production was not affected (Fig. 5g).

**Figure 6:**
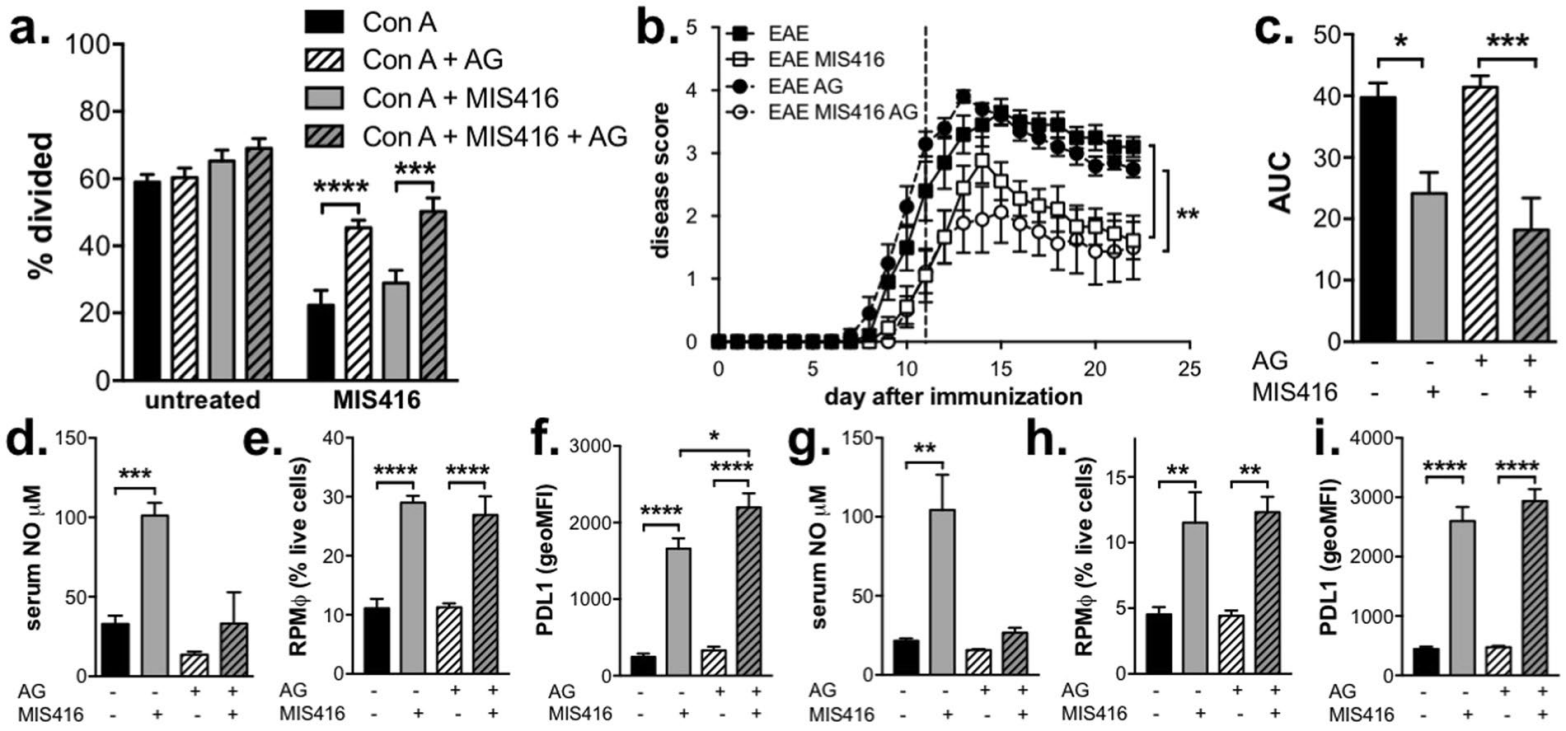
Inhibition of NO restored CD4 T cell proliferation but did not alter the ability of MIS416 to inhibit EAE or modulate spleen myeloid subsets. (**a**) Splenocytes (1 × 10^6^/ml) were isolated from mice 15 days post MIS416 treatment initiation, labelled with CSFE, and stimulated *in vitro* with Con A (3 μg/ml) +/− MIS416 (20 μg/ml) in the presence of absence of aminoguanidine (AG; 200 mM) for 48 hours. The % of CD4^+^ cells that divided was quantified by flow cytometry. Shown are the means and SEM of individual mice (n = 8–10/group) from 2 independent experiments. ***p < 0.001 and **p < 0.01 by two-way ANOVA with Sidak’s multiple comparison test. (**b**,**c**) Mice were treated with MIS416 weekly (i.v.; 100 μg/mouse) starting on day 0, immunized to induce EAE, and scored daily for EAE (**b**) with the cumulative disease shown as area under the curve (AUC; **c**). Aminoguanidine (AG; 100 mM) was administered in the drinking water starting on day 12 (dotted line). Shown are the means and SEM of individual mice (n = 9–10/group) from 2 independent experiments. ***p < 0.001 and *p < 0.05 by two-way ANOVA with Sidak’s multiple comparisons test (**b**) and ***p < 0.001 and *p < 0.05 by one-way ANOVA with Tukey’s multiple comparisons test. (**d–f**) Splenocytes and serum were isolated from EAE mice treated with MIS416 (weekly; starting day 0) and/or AG (100 mM; starting day 12) 22 days post immunization. Serum NO (**d**) was assessed directly, and the total number of red pulp macrophages (RPMφ; **e**) and expression of PDL-1 (geoMFI; **f**) was determined by flow cytometry. Shown are the means and SEM from individual mice (n = 4–5/group) from one experiment. (**g–i**) Splenocytes and serum were isolated from healthy mice treated with MIS416 and/or AG (100 mM; starting day 0) 15 days post MIS416 treatment initiation. Serum NO (**g**) was measured directly, and the total number of red pulp macrophages (RPMφ; **h**) and expression of PDL-1 (geoMFI; **i**) were determined by flow cytometry. Shown are the means and SEM from individual mice from two independent experiments (**h**; n = 7–8/group), and one experiment (**i**; n = 5/group). (**d–i**) ****p < 0.0001, ***p < 0.001, **p < 0.01 and *p < 0.05 by one-way ANOVA with Tukey’s multiple comparisons test.

NO plays a role in both EAE disease development and disease pathogenesis; the timing of NO induction being a determinant for whether this molecule acts to enhance or suppress EAE disease^16–19^. Since NO can act to suppress T cell proliferation^18^, we determined whether MIS416-induced NO was involved in the suppression of EAE. Inhibition of iNOS, the enzyme responsible for NO synthesis, by addition of aminoguanidine to splenocyte cultures from MIS416-treated mice reduced the suppression of CD4 T cell proliferation but did not fully rescue T cell proliferation indicating that MIS416 may exert additional effects on T cells (Fig. 6a). To understand the effect of MIS416-induced NO *in vivo*, aminoguanidine was administered in the drinking water from the onset of EAE (day 12), but was not found to alter the disease-protective effects of MIS416 (Fig. 6b,c) despite reducing serum NO levels (Fig. 6d). Interestingly, inhibition of NO *in vivo* did not affect the expansion of red pulp macrophages nor the upregulation of PDL-1 on splenic myeloid cells in MIS416-treated immunized (Fig. 6d–f) or healthy mice (Fig. 6g–i). Taken together, these results indicate that while NO may contribute to the observed suppression of T cell proliferation by MIS416, it is likely that the disease protective effects in EAE are due to other IFN-γ-mediated effects.

### MIS416 reduced CNS invasion by immune cells during EAE while promoting the homeostatic trafficking of PDL-1-expressing myeloid populations in an IFN-γ-dependent manner

**Figure 7:**
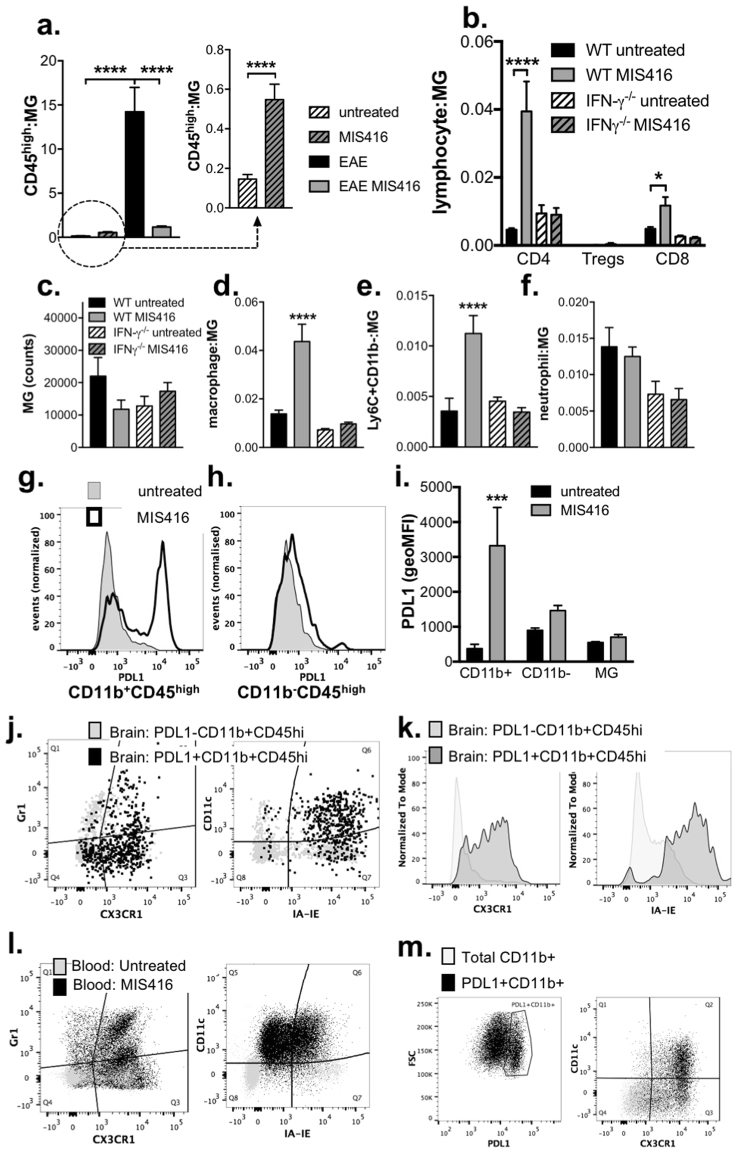
MIS416 reduced immune cell invasion during EAE but promoted the recruitment of PDL-1 expressing immune cells into the CNS in an IFN-γ-dependent manner in healthy mice. (**a**) CD45^+^ cells were isolated from the spinal cords of MIS416-treated and untreated EAE mice 22 days post immunization and healthy, MIS416-treated and untreated mice 15 days post treatment initiation. Gating strategy is shown in Suppl Fig. 4 and data expressed as the ratio of CD45^high^ cell: microglia (CD45^int^CD11b^+^). Shown are the means and SEM of individual mice (n = 23/EAE and 11/healthy group) from 3 independent experiments/group. ****p < 0.0001 by one-way ANOVA with Tukey’s multiple comparison test and ****p < 0.0001 by unpaired Student’s t test (inset). (**b–k**) CD45^+^ cells were isolated from the brains of healthy, MIS416-treated and untreated WT and IFN-γ^−/−^ mice 15 days post MIS416 treatment initiation and assessed by flow cytometry. Gating strategies are shown in Suppl Fig. 4 (T cells) and 6 (myeloid cells). **(b)** CD4^+^ and CD8^+^ cell results (mean and SEM) are from individual mice (n = 7–11/group) from 3 independent experiments. ****p < 0.0001 and * p < 0.05 by one-way ANOVA with Sidak’s multiple comparison test (untreated vs MIS416). **(c–f**) Myeloid cell results (mean and SEM) are from individual mice (n = 6–7/group) from 2 independent experiments. ****p < 0.0001 and ***p < 0.001 by one-way ANOVA with Sidak’s multiple comparison test (untreated vs MIS416). (**g–k**) MIS416 treatment enhanced PDL-1-expression on CD11b^+^CD45^high^ (**g**) but not CD11b^-^CD45^high^ cells (**h**) or microglia (**i**) in the brain. (**j**,**k**) The PDL-1^+^CD11b^+^CD45^high^ cells in the brain had a restricted myeloid phenotype characterized by increased expression of CX3CR1, IA-IE, and CD11c compared to PDL-1^-^CD11b^+^CD45^high^ cells. (**l**,**m**) MIS416 increased the proportion of inflammatory and non-inflammatory monocytes in the blood and PDL1 + cells that preferentially expressed CX3CR1 and CD11c. Shown are representative flow plots of gated CD45+ cells from the brains (**g**–**k**) or peripheral blood (**l**,**m)** from untreated and MIS416-treated healthy mice 15 days post MIS416 treatment initiation. (**i**) Shown are the means and SEM of individual mice (n = 4/group) from 1 of 2 independent experiments. ***p < 0.001 by two-way ANOVA with Sidak’s multiple comparison test (untreated vs MIS416).

Recent work has shown that IFN-γ promotes trafficking of regulatory immune cells^20^ into the CNS through the choroid plexus^21^, a point of entry that is predominantly associated with innate immune surveillance and the maintenance of CNS homeostasis. Thus, we assessed the extent to which MIS416 altered CNS leukocyte trafficking during EAE-associated inflammation and also determined whether MIS416 promoted IFN-γ-dependant homeostatic trafficking to the CNS of healthy mice. As expected, MIS416-mediated attenuation of EAE was associated with a significant reduction in the number of CD45^high^ cells in the spinal cords during EAE (Fig. 7a and Suppl Figs 5 and 6), including a significant reduction in CD4^+^ and CD8^+^ T cell populations (Suppl Fig. 5b). In contrast, MIS416 treatment of healthy mice led to an increase in these cells in the spinal cord (Fig. 7a; see inset) and brain (Fig. 7b–f). The increase in CD45^high^ cells in the brains of MIS416-treated healthy animals consisted primarily of CD4 T cells, macrophages and a Ly6C^+^CD11b^low^ monocytic myeloid population (Fig. 7b,d,e and Suppl Figs 5 and 7). Significantly, this recruitment was IFN-γ-dependent (Fig. 7b,d, and e), and leukocyte subset specific as MIS416 treatment did not lead to an increase in Treg, neutrophils, or microglia (Fig. 7b,c,f). Lastly, the expression of PDL-1 that was shown to be significantly upregulated on peripheral myeloid cells was also highly expressed on the peripheral myeloid subset (CD11b^+^CD45^high^) that were recruited to the brain by MIS416 treatment. Analysis of markers that discriminate non-inflammatory/non-classical blood monocytes from their pro-inflammatory/classical counterparts demonstrated these cells had the restricted phenotype of non-inflammatory monocytes cells with upregulation of markers associated with myeloid activation/macrophage maturation (CX3CR1^+^, CD11c^+^, and IA-IE^+^; Fig. 7g–k) similar to their CD11b^−^CD45^high^ peripheral blood counterpart (Fig. 7l,m). Overall, these results suggest that MIS416 may provide protection from EAE by driving innate IFN-γ production, which enhances homeostatic recruitment of PDL-1-expressing myeloid cells to the CNS, where they may establish a more immune regulatory environment that restricts neuroinflammation.

## Discussion

The innate immune system is thought to play a pivotal role in MS pathogenesis, particularly in progressive disease; therefore, targeting innate cell receptors has recently been recognised as a potential therapeutic strategy^22^. Treatment with a NOD-2 and TLR9 agonist, MIS416, has shown success for reducing disease burden in the EAE model when given prophylactically and therapeutically. This protection depended on IFN-γ, a cytokine that was also significantly increased in MIS416-treated secondary progressive MS patients^4^. The dose of MIS416 that effectively reduced EAE disease also induced a number of immunological changes within the innate and adaptive immune compartments which may contribute to its disease-modifying activity. The current study aimed to determine which of these immune alterations were orchestrated by IFN-γ and evaluate their contribution to the mechanism of action of MIS416 in EAE.

It was determined that MIS416 treatment significantly reduced antigen-specific and non-specific T cell responses *in vitro* and *in vivo*, however in the absence of IFN-γ this capability of MIS416 to suppress T cells was lost. Additionally, the absence of MIS416-induced IFN-γ abrogated myeloid cell expansion and expression of NO and PDL-1, both of which are likely to contribute to suppressed T cell responses and ultimately, reduced EAE disease^18,23^. While NO was found to have an important function in suppressing T cell proliferation *in vitro*, depleting NO *in vivo* did not affect MIS416 disease protection. This result indicates that other factors such as myeloid expression of PDL-1 are likely to have a more central role in the mechanism underpinning suppressed T cell responses *in vivo*.

Central to immune homeostasis, the ligand PDL-1 is expressed by many different cell types, including both hematopoietic and non-hematopoietic cells, whereas expression of PDL-2 is limited to activated antigen presenting cells such as macrophages and dendritic cells. The engagement of PDL-1/PDL-2 with its receptor, PD-1, on T cells maintains homeostasis by limiting T cell responses during infection and preventing autoimmunity^24^. The PD-1/PDL-1 pathway has been extensively studied recently in the context of cancer immunology as the expression of PDL-1 in the tumour microenvironment is thought to be one of the mechanisms by which cancer cells suppress anti-tumour T cell responses and evade recognition by the host immune system. More recently, clinical trials have shown that treatment with antibodies that block T cell PD-1 signalling, such as Nivolumab, successfully initiate anti-cancer immune responses and increase patient survival^25^. Increased PDL-1 expression has been shown to be important in naturally limiting EAE disease, as work by Latchman et al. showed that PD-L1^−/−^ mice have enhanced T cell responses *in vitro* and develop more severe EAE than wild type mice^26^. In addition, PDL-1 expression was increased during the acute phase of disease indicating its role in limiting disease severity^27^. Other studies investigating EAE in PD-1^−/−^ and PDL-1^−/−^ mice have identified that the absence of PD-1 signalling Th1 and Th17-type cytokines were increased^23^. Such studies provide strong evidence that the PDL-1/PD1 axis is a pivotal check point that determines T cell fate and support the hypothesis that MIS416-mediated upregulation of the PDL-1 pathway is a key mechanism by which MIS416 reduces the severity of EAE. Notably, enhanced myeloid cell expression of PDL-1 is also associated with recombinant IFN-β treatment, an approved therapy for treating relapse remitting MS^28,29^. The activation of the Type I/II interferon axis by both recombinant IFN-β and MIS416, further highlights the significance of the PDL-1-Type I/II interferon axis to the proposed mechanism of action of MIS416.

In addition to its effects on T cell anergy and apoptosis, PDL-1 expression may be protective in EAE through the induction of Treg. Several studies have shown that the expression of PDL-1 on myeloid cells induces the formation of Treg as well as maintains the suppressive nature of the Treg population^30,31^. In agreement with these findings, we found MIS416-treated mice had increased Treg number and suppressive function in conjunction with increased PDL-1 expression. However, in the absence of IFN-γ while there was very little myeloid PDL-1 expression, Treg expansion still occurred, albeit to a lesser extent, suggesting that Treg expansion alone was not responsible for disease protection.

In addition to suppressed peripheral T cell responses and reduced inflammatory cell trafficking to the CNS, MIS416 was demonstrated to induce IFN-γ-dependent recruitment of regulatory PDL-1^+^ myeloid cells into the CNS in the absence of neuroinflammation - another mechanism by which MIS416 may exert its effects *in vivo*. The concept that IFN-γ can enhance CNS-trafficking^20^ of non-inflammatory myeloid cells through the choroid plexus is well supported^21,32,33^. Such PDL-1 expressing myeloid cells may be able to suppress T cell activity within the CNS, directly reducing CNS inflammation and recruitment of pathogenic leukocytes from the periphery.

In summary, this work has uncovered a unique mechanism by which an innate-myeloid targeted therapeutic can selectively modulate CNS-trafficking and reduce neuroinflammation. We have found that this mechanism is dependent upon IFN-γ, which appears to mediate its protective actions through myeloid cell expansion and PDL-1 upregulation. This finding is particularly significant as MIS416 has been shown to induce elevated serum IFN-γ in secondary progressive MS patients treated with MIS416^4^. Having shown clinical promise during preliminary trials^2,3^, MIS416 is currently completing a phase 2b exploratory trial in secondary progressive MS. This work provides critical insight into how this novel MS therapeutic may reduce compartmentalized neuroinflammation that occurs in progressive MS. MIS416 has the capacity to favour CNS recruitment of myeloid cells associated with anti-inflammatory/tissue repair activity in the presence of an intact blood barrier and represents the first example of an MS therapeutic that can amplify peripheral, regulatory myeloid cells and licence their trafficking to the CNS^34,35^.

## Electronic supplementary material

Supplementary Figures
