# Supplementary material for: Innate IFN-γ ameliorates experimental autoimmune encephalomyelitis and promotes myeloid expansion and PDL-1 expression
Source: Sci Rep. 2018 Jan 10;8:259. doi: 10.1038/s41598-017-18543-z (PMC5762891; doi:10.1038/s41598-017-18543-z)
Supplement: Supplementary file 1 — Supplementary Figures [file 41598_2017_18543_MOESM1_ESM.pdf]

Innate IFN-γ ameliorates experimental autoimmune encephalomyelitis and promotes myeloid expansion and PDL-1 expression.

Madeleine P. J. White[1], Gill Webster[2], Faith Leonard[1,2], Anne Camille La Flamme[1,3]

[1]School of Biological Sciences, Victoria University of Wellington, Wellington, New Zealand; [2]Innate Immunotherapeutics, Auckland, New Zealand; and [3]Malaghan Institute of Medical Research, Wellington, New Zealand

Corresponding author: Prof Anne C. La Flamme, School of Biological Sciences, Victoria University of Wellington, P.O. Box 600, Wellington, Wellington, New Zealand (tel) +64-4-463-6093 (fax) +64-4-463-5331 (e-mail) anne.laflamme@vuw.ac.nz

Supplementary data

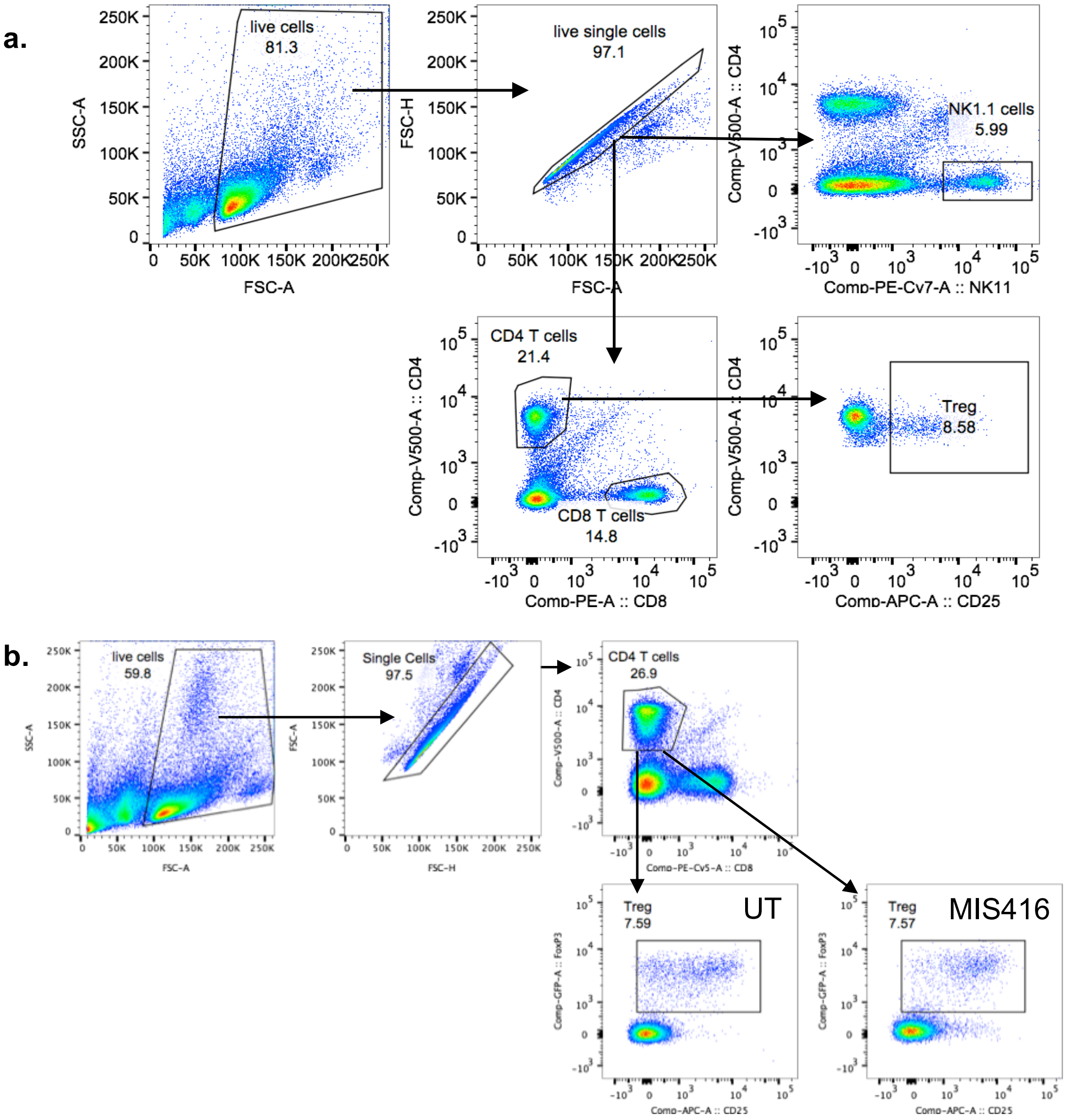

Supplementary Figure 1: Gating for splenic lymphocytes. **(a)** C57BL/6 mice were treated with MIS416 (100 μg/mouse weekly i.v.) or untreated (UT) as described in Figure 1c. Fifteen days post-immunization, splenocytes were isolated and analyzed by flow cytometry. Shown are representative flow plots illustrating the gating strategy for CD4 T cells, CD8 T cells, and CD25$^+$CD4$^+$ T cells (Tregs). **(b)** To verify expression of FoxP3 by CD25$^+$CD4$^+$ Tregs, FoxP3-GFP mice were treated with MIS416 or UT as above and 15 days later, splenocytes were analyzed by flow cytometry. Shown are representative flow plots demonstrating FoxP3 expression on CD25$^+$CD4$^+$ Tregs from MIS416 and UT mice.

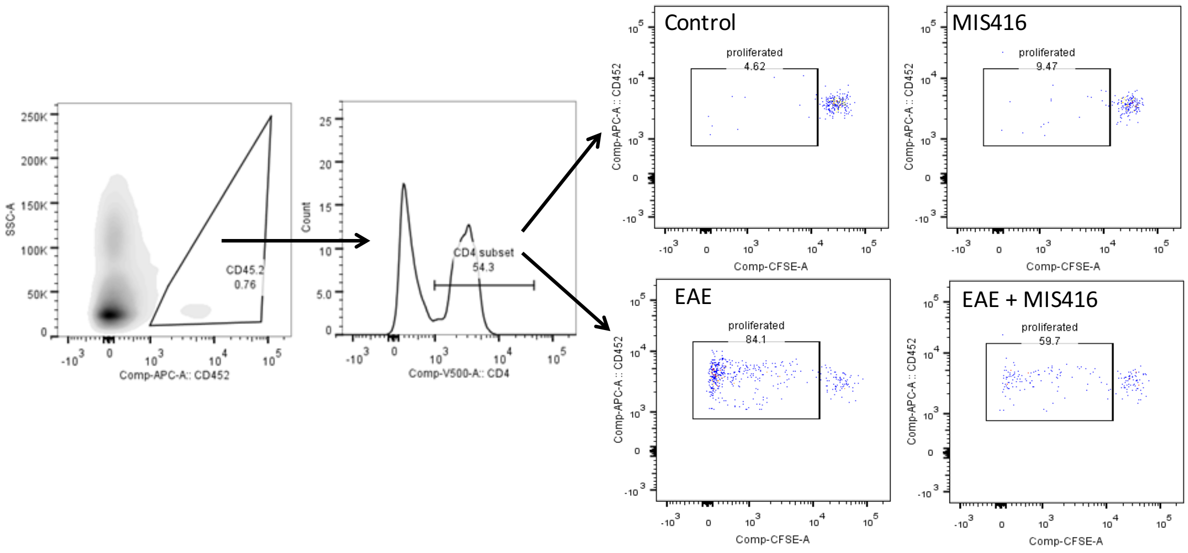

<u>Supplementary Figure 2: Gating strategy for assessing *in vivo* proliferation.</u> CFSE-labelled 2D2 cells were transferred into CD45.1 congenic mice one day before MIS416 treatment and EAE immunization. After 5 days, proliferation of CD45.2$^+$ 2D2 CD4 T cells in the draining lymph nodes and spleens was assessed by flow cytometry. Shown is the gating strategy to identify proliferating CD45.2$^+$ 2D2 CD4 T cells.

a.

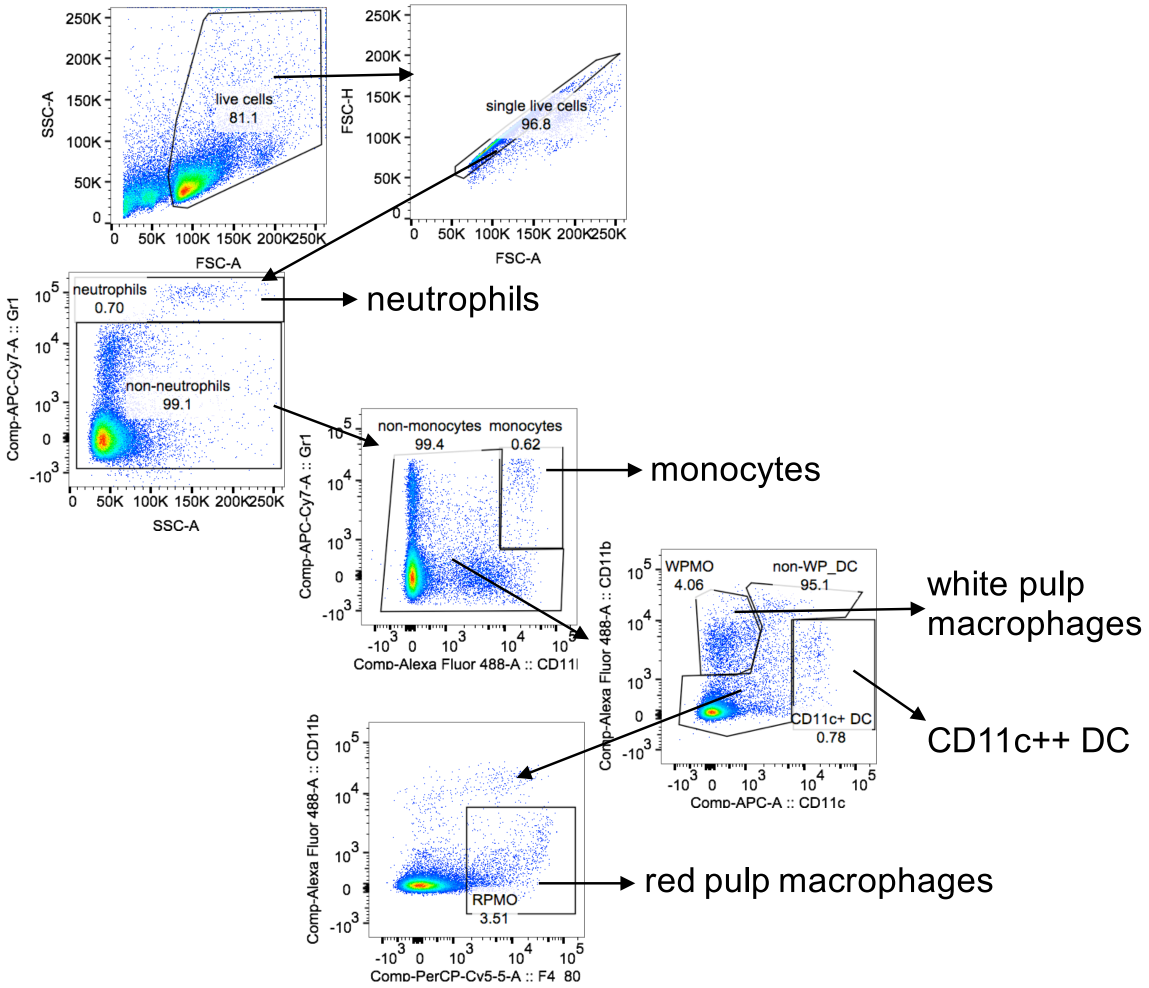

b.

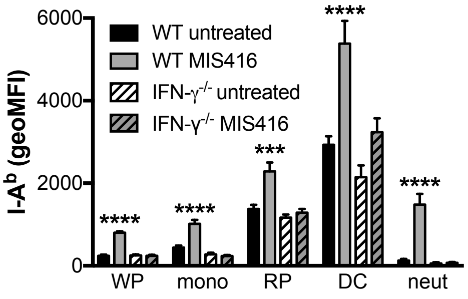

Supplementary Figure 3: (**a**) Gating strategy for splenic myeloid populations and (**b**) I-A^b expression on splenic myeloid populations. Splenocytes were isolated from WT C57BL/6 and IFN-γ$^{-/-}$ mice 15 days post MIS416 treatment initiation, and the splenic myeloid subsets (**a**) and expression of I-A^b (**b**; geoMFI) were assessed by flow cytometry. Shown are the gating strategy for the myeloid subsets (**a**) and the means and SEM from individual mice (n = 4 and 7 mice/group, WT and IFN-γ$^{-/-}$, respectively) from two experiments (**b**). **** $p < 0.0001$ and *** $p < 0.001$ by one-way ANOVA with Tukey's multiple comparisons test.

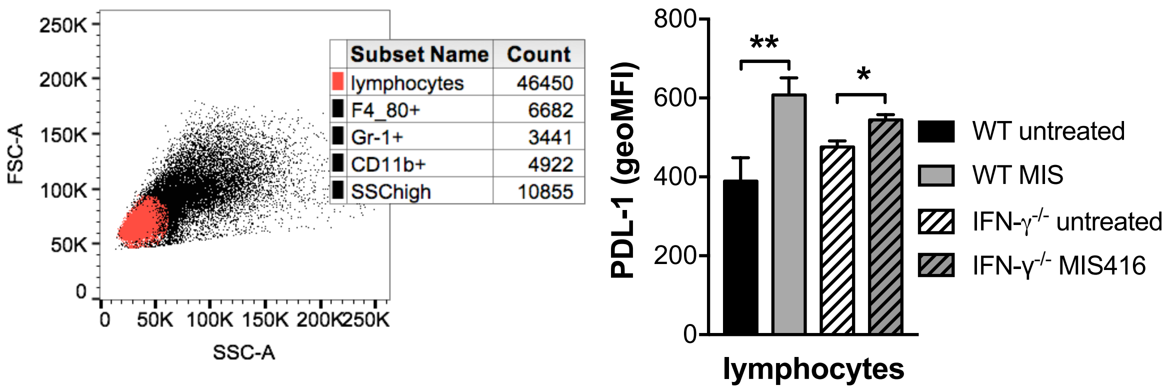

Supplementary Figure 4: MIS416 increased PDL-1 expression on lymphocytes. Splenocytes were isolated from WT C57BL/6 and IFN-$\gamma^{-/-}$ mice 15 days post-MIS416 treatment initiation and analyzed by flow cytometry. (**a**) The lymphocyte population (identified as SSC^{low}, $CD11b^{-}$, $Gr\text{-}1^{-}$, and $F4/80^{-}$) had a distinct forward and side scatter (red) profile to myeloid cells (black). (**b**) Expression of PDL-1 on lymphocytes was enhanced by MIS416 treatment even in the absence if IFN-γ. Shown are the gating strategy for lymphocytes (**a**) and the means and SEM from individual mice (n = 9-11 mice/group) from three experiments (**b**). ** $p < 0.01$ and * $p < 0.05$ by one-way ANOVA with an uncorrected Dunn's test.

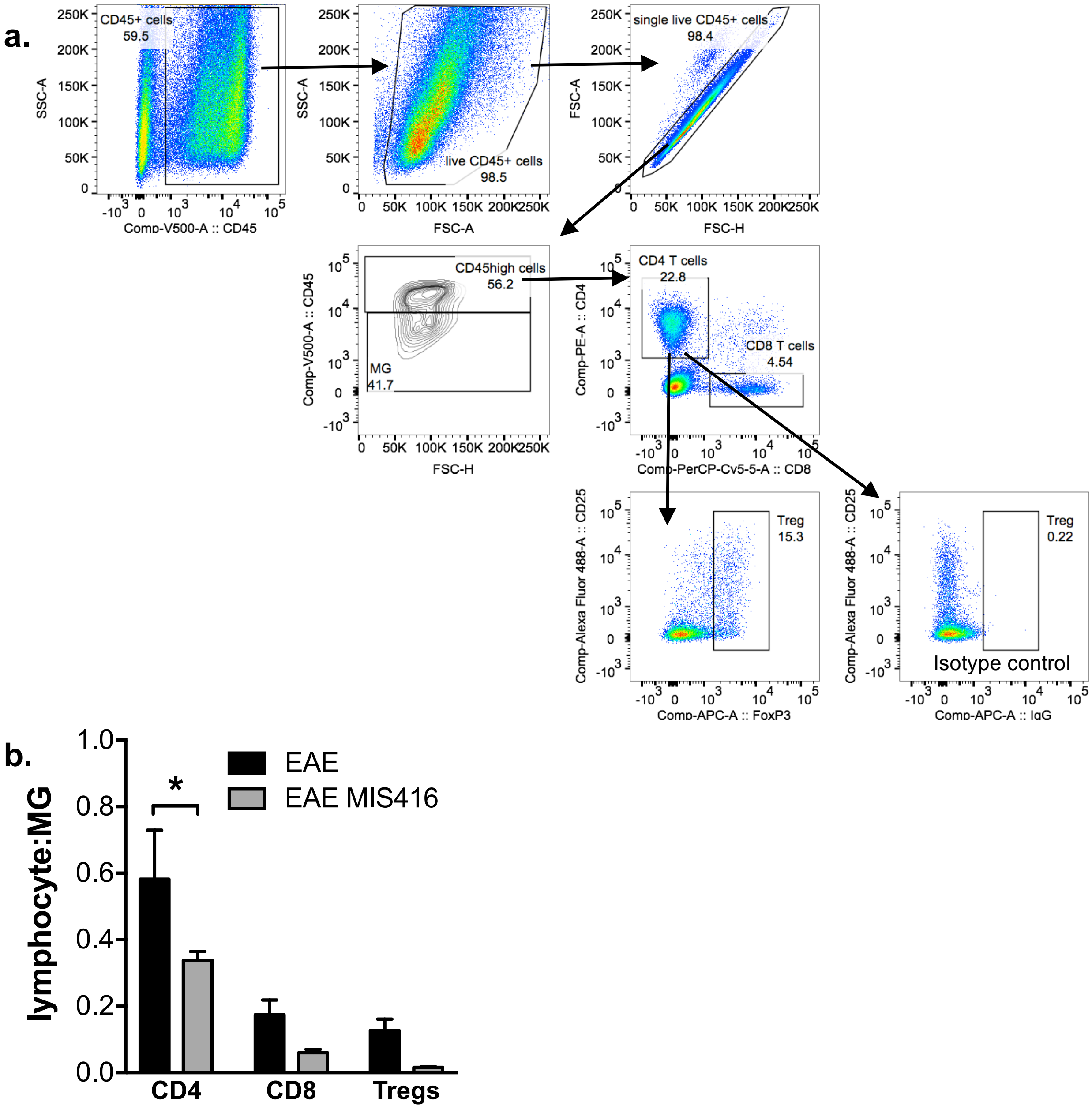

Supplementary Figure 5: T cell CNS populations CD45^{+} cells were isolated from the spinal cords of C57BL/6 mice immunized to induce EAE 22 days previously and treated weekly with MIS416 (100 μg/mouse i.v.). (**a**) Representative flow plots from an untreated EAE mouse illustrating the gating strategy for the T cell subsets. (**b**) Data are expressed as the ratio of CD45^{high} T cell:microglia (CD45^{int}CD11b^{+}). Shown are the means and SEM from individual mice (n = 8 mice/group) from one of two representative experiments. * $p < 0.05$ by two-way ANOVA with Sidak's multiple comparisons test.

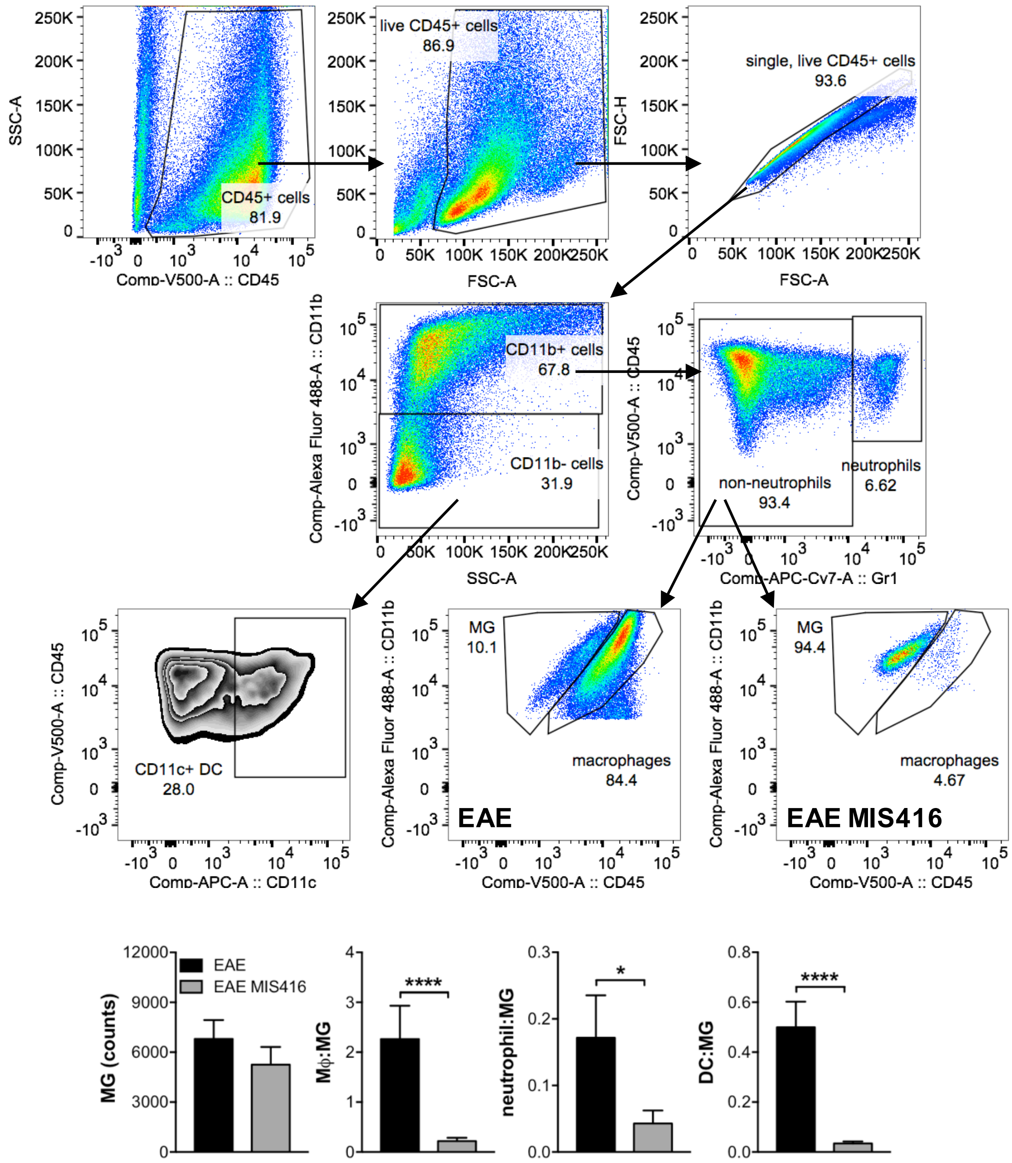

Supplementary Figure 6: Spinal cord myeloid populations. $CD45^+$ cells were isolated from the spinal cords of C57BL/6 mice immunized to induce EAE 22 days previously and treated weekly with MIS416. **(a)** Shown are representative flow plots illustrating the gating strategy to identify myeloid populations. The same strategy was used for CD45+ cells in the brain during EAE. **(b)** Data are expressed as the ratio of $CD45^{high}$ myeloid cells:microglia ($CD45^{int}CD11b^+$). Shown are the means and SEM from individual mice (n = 13 mice/group) from 13 independent experiments. * p < 0.05 and **** p < 0.0001 by Mann-Whitney test.

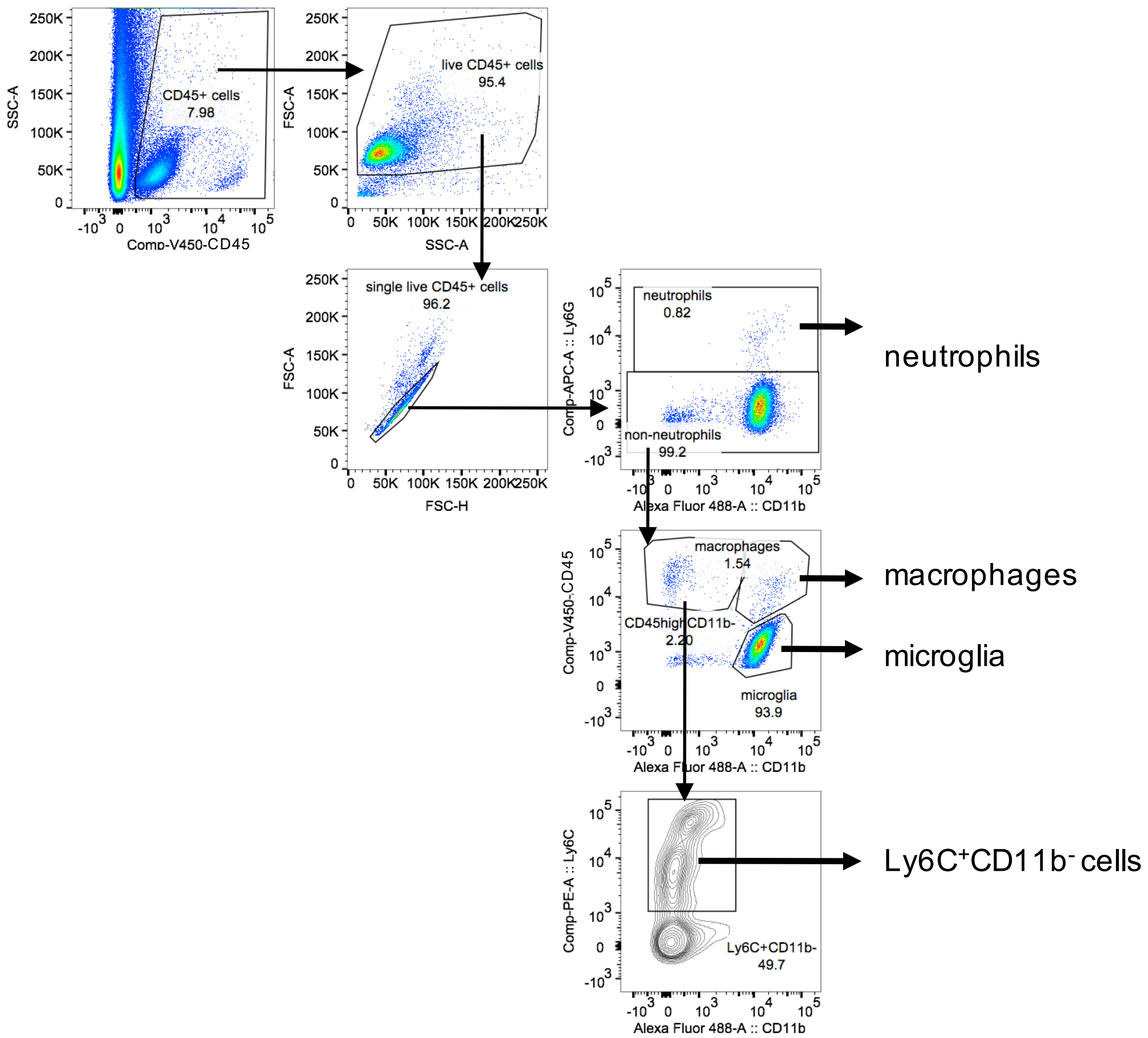

Supplementary Figure 7: Brain myeloid populations. CD45$^+$ cells were isolated at day 15 from the brains of healthy C57BL/6 mice treated weekly with MIS416. **(a)** Shown are representative flow plots from an MIS416-treated WT mouse illustrating the gating strategy to identify myeloid populations in the brain.
